# Population Genomics and the Statistical Values of Race: An Interdisciplinary Perspective on the Biological Classification of Human Populations and Implications for Clinical Genetic Epidemiological Research

**DOI:** 10.3389/fgene.2016.00022

**Published:** 2016-02-17

**Authors:** Koffi N. Maglo, Tesfaye B. Mersha, Lisa J. Martin

**Affiliations:** ^1^Department of Philosophy, Center for Clinical and Translational Science and Training, University of CincinnatiCincinnati, OH, USA; ^2^Department of Pediatrics, Cincinnati Children's Hospital Medical Center, University of CincinnatiCincinnati, OH, USA

**Keywords:** ancestry, cline, cluster analysis, Darwinian classification, genomic medicine, pair-wise *F*_*st*_, phylogenomics, population structure

## Abstract

The biological status and biomedical significance of the concept of race as applied to humans continue to be contentious issues despite the use of advanced statistical and clustering methods to determine continental ancestry. It is thus imperative for researchers to understand the limitations as well as potential uses of the concept of race in biology and biomedicine. This paper deals with the theoretical assumptions behind cluster analysis in human population genomics. Adopting an interdisciplinary approach, it demonstrates that the hypothesis that attributes the clustering of human populations to “frictional” effects of landform barriers at continental boundaries is empirically incoherent. It then contrasts the scientific status of the “cluster” and “cline” constructs in human population genomics, and shows how cluster may be instrumentally produced. It also shows how statistical values of race vindicate Darwin's argument that race is evolutionarily meaningless. Finally, the paper explains why, due to spatiotemporal parameters, evolutionary forces, and socio-cultural factors influencing population structure, continental ancestry may be pragmatically relevant to global and public health genomics. Overall, this work demonstrates that, from a biological systematic and evolutionary taxonomical perspective, human races/continental groups or clusters have no natural meaning or objective biological reality. In fact, the utility of racial categorizations in research and in clinics can be explained by spatiotemporal parameters, socio-cultural factors, and evolutionary forces affecting disease causation and treatment response.

## Introduction

While pervasive, the concept of race is both a problematic and highly misunderstood concept in biological and biomedical research (Smedley and Smedley, 2005). This is not new, and for centuries, the debate on whether biological human races exist has raged (Risch et al., 2002; Burchard et al., 2003; Ossorio and Duster, 2005; Caspari, 2009; Frank, 2014; Guo et al., 2014; Duster, 2015). The completion of the Human Genome Project seems to have added fuel to the ongoing debate. Indeed, when looking at continental ancestry, a relatively small number of genetic markers can separate populations into meta-populations (Paschou et al., 2008; Nelis et al., 2009). On the other hand, when examining larger number of markers, there is a tremendous diversity within groups (Hunley et al., 2009; Baye et al., 2011).

The relevance of racial classifications to biomedical research is also unclear. There are many examples in the literature where racial differences in health related phenotypes exist (e.g., obesity, asthma, and breast cancer; Ogden et al., 2012; Romero et al., 2012; Howlader et al., 2014b; Keet et al., 2015). However, the predictive ability of race in epidemiological and clinical research is generally weakened by potential confounders. Yet, the mandate by funding agencies like the National Institutes of Health on capturing racial information of research participants ensures that race cannot be ignored (Stevens, 2003; Maglo and Martin, 2012; Bliss, 2013; Maglo et al., 2014). Thus it is crucial for researchers and clinicians to understand the issues surrounding the biological status and biomedical significance of the concept of race (Maglo, 2010, 2012; Mersha and Abebe, 2015).

The purpose of this paper is to describe the scientific basis of the concept of race in biological systematic and evolutionary classification and its applications in current biomedical research by reviewing and evaluating the current literature. Specifically, we will scrutinize the issue of the biological basis by discussing the phylogenetic and evolutionary criteria for the objective existence or natural reality of biological groupings/taxa of organisms. We will apply genetic data to evaluate the evidence for the putative existence of biological human races. Lastly, we will consider the implications and utility of racial grouping to biomedical research. Taken together, this paper demonstrates that, despites technical and technological advances in clustering methods, cline remains the foundational concept in human population genomics, that continental clusters are merely instrumentally produced, and that human races/continental groups have no natural meaning or objective reality from a biological systematic and evolutionary taxonomical perspective. There are gradations (clines) in human population genetic profiles, and without understanding allelic distributions across human populations and their practical biomedical implications, the potential for reification and misinterpretation of racial disparities in epidemiological and clinical research is great.

## Phylogenetic and evolutionary criteria for biological classification

There are two evolutionary theoretical criteria for naturally objective groupings of biological organisms. These are common ancestry and degree of similarity (Mayr and Bock, 2002; Schuh and Brower, 2009; Wiley and Lieberman, 2011; Templeton, 2013). Phylogenetic systematics and Darwinian/evolutionary taxonomy use “common descent” as a criterion for biological classification but the similarity criterion is used only in the latter. Systematics and evolutionary classification are concerned with organic diversity and evolutionary relationships. The assumptions underlying the primary use of neutral markers in human genetic diversity studies suggest that their objective biological meaning needs to be evaluated based on the above two criteria. Yet as researchers increasingly point out, the debate is “free floating” to the extent that what counts as “biological reality” of human races is elusive, ranging from “trivial” to “obscure,” and often construed in a non-Darwinian biological framework (Cavalli-Sforza, 2000; Cooper et al., 2003; Graves, 2011; Maglo, 2011).

The mounting questionable assumptions underlying biological race theories have recently led scholars to remind the research community about Dobzhansky's (1973) paper tellingly entitled “Nothing in biology makes sense except in light of evolution” (Dobzhansky, 1973; Graves, 2011). In fact, although biologists and ordinary people are interested in various forms of classifications of biological entities, not every classification reflects an evolutionary ordering of living things (Dupré, 1993; Mayr and Bock, 2002). Phylogenetic systematics, for one, posits that the various other types of biological relationships researchers are concerned with, including ecological relationships and similarity, “have maximum relevance when understood within the context of genealogical descent” (Wiley and Lieberman, 2011).

Darwinian or evolutionary classification on its part deploys the two criteria for ordering organisms, discussed above, and is also different even from biological classifications “of cell, tissue and organ types of different groups of organisms, of ecological communities, of behavioral activities and so forth” (Mayr and Bock, 2002). While the objectivity of evolutionary kinds, understood as evolutionary ordered taxa of organisms, is defined either by common descent or genetic similarity, the similarity itself is construed as deriving from homologous characteristics due to shared ancestry rather than deriving from homoplastic characteristics due to parallelism, convergence, or reversal (Mayr and Bock, 2002; Fujimura and Rajagopalan, 2011).

Accordingly, a taxon of organisms may be said to have an objective independent biological existence in Darwinian classification if either of the following two conditions obtains: (1) It constitutes a phylognetic clade by comprising all, but only all, the descendants of its originating biological common ancestor (Templeton, 1998, 2013; Schuh and Brower, 2009; Claridge, 2010; Mishler, 2010; Maglo, 2011; Wiley and Lieberman, 2011); and/or (2) It has reached a degree of genetic differentiation deemed taxonomically meaningful in system biology (Mayr and Bock, 2002; Keita et al., 2004; Graves, 2011). Thus, it follows from these evolutionary theoretical constraints that races must be evolutionary distinct human subpopulations by virtue of (1) or (2) or some combination of both in order to be a valid biological category.

In Darwinian classification (but also in phylogenetic systematics), a biological grouping of organisms that does not meet the above criteria is referred to as a wastebasket taxon. It is so called because it is evolutionary unordered and functions in science merely as a “warehouse kind” that taxonomically lumped together disparate organisms having no objectively definable evolutionary relationship. Wastebasket taxa lack natural reality (Parfrey et al., 2006; Schuh and Brower, 2009; Claridge, 2010; Mishler, 2010; Wiley and Lieberman, 2011) and granting them objective biological existence constitutes an erroneous attribution of ontological status called the fallacy of reification (Gannett, 2004, 2014; Duster, 2005; Glasgow, 2009; Maglo and Martin, 2012; Hochman, 2013).

### Hierarchical population structure, clusters, and race

**Figure 1:**
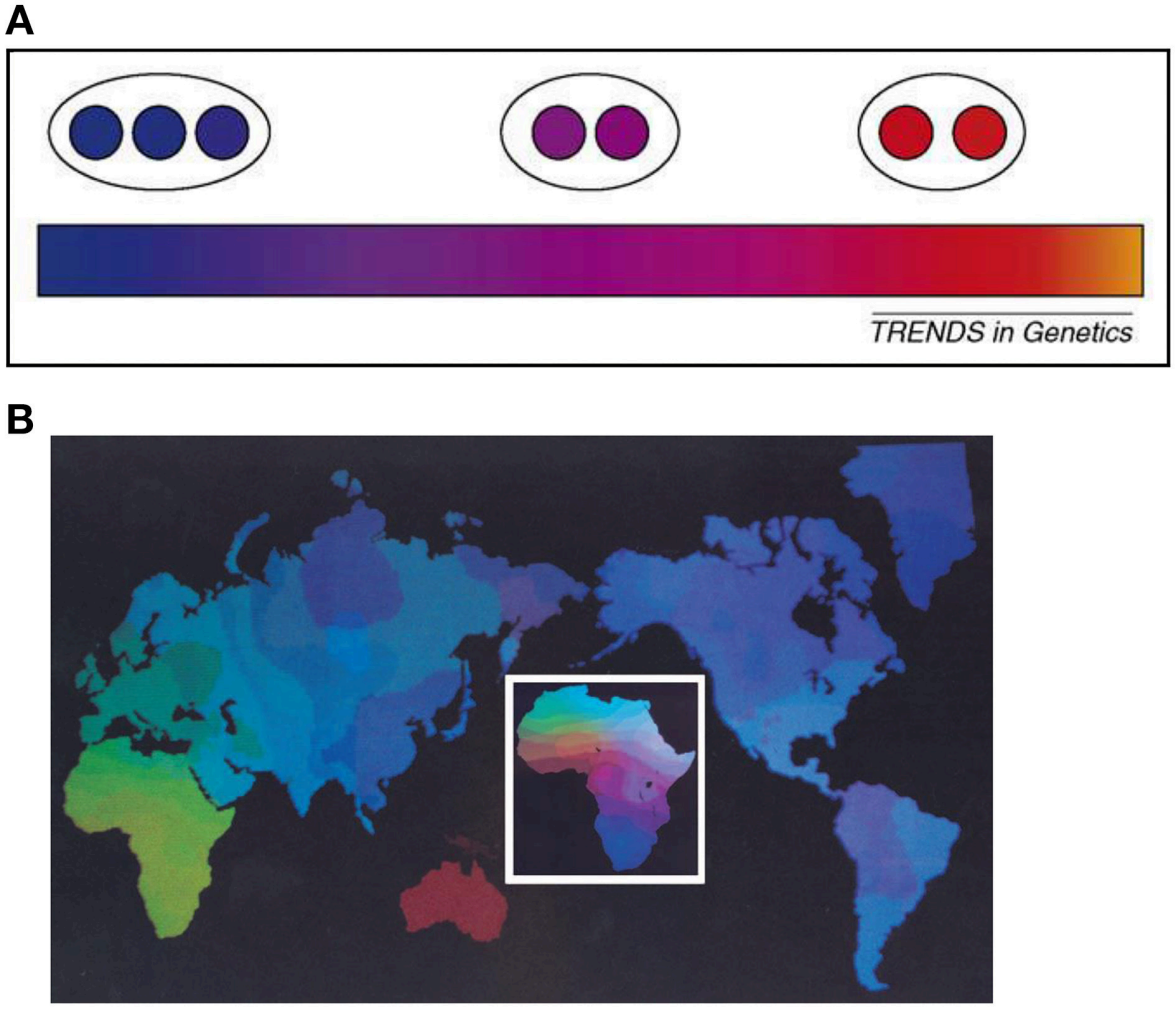
**(A)** The effect of sampling strategies (adopted from Handley et al. 2007) [“Heterogeneous sampling can reveal genetic clusters that are biologically meaningless. The gradation in color from blue to orange represents a hypothetical situation of strictly continuous variation in allele frequencies. If sampling is heterogeneous (population samples represented here by circles) then the pattern of clinal variation can be mistaken for genetically distinct clusters (black ellipses)”] (Handley et al., 2007). **(B)** Global genetic diversity in humans are distributed in gradients among and within continents, emphasizes intercontinental variation. The inset figure of Africa highlights finer gradations over shorter geographic spans, emphasizing the “clinality” of human genetic variation (adopted from Cavalli-Sforza et al., 1994). Figure reproduced with permission from Cell Press/Elsevier.

One way to explain the reification problem in the biological classification of organisms is to consider the hierarchical population structure model which would seem to lend support to the identification of distinct clusters of human populations. For the cluster approach, one defines distinct subgroups from the genetic substructure within a population, (Figure 1A). Under the hierarchical population structure model, a population is made up of subpopulations deriving from a fragmentation history. Fragmentation may be due to various causes including habitat fragmentation which itself may be induced by natural factors (geological and climatic) or human factors (social and cultural). Population fragmentation engenders substructures and may lead to the emergence of meta-populations (Table 1). The total genetic variation in the population is then the sum of the genetic variation of the fragmented subpopulations (Relethford, 2012). But a population with a history of fragmentation may have various divisionary levels (DLs), i.e., structural layers (Maglo, 2010, 2012).

**Table 1:** **Illustration of the concept of divisionary level (DL) with three continental groups**.

**Divisionary Levels (DL)**	**Populations**		
DL-1	**Human species**		
DL-2	Africans	Eurasians	East Asians
DL-3	West Africans	Europeans	Han Chinese
DL-4	Sahelians	North Europeans	Southern Han Chinese
DL-5	Mandinka	Swedish	Yue Chinese
DL-6	Malian Mandinka	Central Swedish	Guangxi Yue Chinese

*It shows putative series of fragmentations of the human species into various subpopulations and the existence of varying degrees of substructures among humans. Yet, DL-2 continental meta-populations do not map natural evolutionary orderings and do not have obvious biological meaning in the rational classification of humans even if they presumably have potential utility. For instance, West Africans are genetically more similar to Europeans than they are to, say, Pygmies with whom they share however the same continental cluster. But although DL-2 clusters mask human evolutionary history, due to environmental factors (including ecological, social, and economic factors), an epidemiological condition may be more or less prevalent among West Africans and Pygmies than Europeans (adapted from Maglo, 2010)*.

For instance, continental clusters are believed to correspond to meta-populations representing the major divisions among humans at the infra-species level. So if one pragmatically takes all humans as a population (divisionary level 1; DL-1), major subgroups within DL-1 will correspond to divisionary level 2 (DL-2). Using the genetic subgroups, DL-2 is reflective of “continental groups” (e.g., Africa, Eurasia, West Asia). But each DL-2 meta-population, may be further subdivided along finer substructures according to the fragmentation of the population history (Cavalli-Sforza et al., 1994; Tishkoff and Verrelli, 2003; Maglo, 2010; Table 1). One of the major factors thought to contribute to fragmentation of populations is geomorphologic barriers constraining population dispersal. For example, the Sahara, Himalayas, and oceans, hereafter SHO, are construed as cluster enabling factors that increase genetic distance between human populations. We shall call this view “the SHO hypothesis.” According to this hypothesis, continental clusters are natural biological groupings because human populations are naturally and distinctly classified based on their genetic differences (Rosenberg et al., 2002, 2005; Rosenberg, 2011).

### Problems with cluster based models of population diversity

One vexing question raised by the steady progress in genomics and computational bioinformatics is how to conceptualize a putative correspondence between race and genomic groupings into continental metapopulations (Cavalli-Sforza, 2000; Glasgow, 2009; Maglo, 2010; Fullwiley, 2011). If we conceive the biological reality or natural character of race as group of organisms in cladistic terms as in criterion (1) above, then phylogenomics supplies the appropriate answer to that question. If continental human populations can be shown to have different recent common ancestors and that each comprises all of the descendants of the respective common ancestor, then the matter can be straightforwardly considered settled according to the cladistic concept of race. However, the problem with cladistic theories of race is that human populations show crisscrossing lineages to the extent that: “A classification that takes into account evolutionary relationships and the nested pattern of diversity would require that Sub-Saharan Africans are not a race because the most exclusive group that includes all Sub-Saharan African populations also includes every non-Sub-Saharan African population…” (Long et al., 2009; Templeton, 2013).

However, cluster analysis is a phenetic method and presupposes, from the perspective of a rational biological classification, the genetic similarity criterion of evolutionary taxonomy. Yet, unlike cluster, it is cline that best accounts for human evolutionary diversity. In fact, the cline model maps continuous genetic gradation in a dataset and indicates that there is no natural break in a population's genetic profile (Figure 1B). Although cluster and cline models are not incompatible, they may lead to competing interpretations. If the population is shown to have a clinal genetic structure but cluster arises in some situations (Ramachandran et al., 2005; Handley et al., 2007; Underhill and Kivisild, 2007), then clustering results cannot be interpreted, in biological taxonomy, as indicative of natural differentiations of biological subpopulations. In this case, cline will be the representation of the natural evolutionary ordering of the population, while cluster will be an artifact, a construct that indicates instrumental, i.e., convenient, cutoff points for various scientific purposes.

## Evaluating the natural character of cluster and cline in human population genomics

There are various ways to measure genetic differences between populations, but perhaps the most popular is the fixation index *F*_*st*_. *F*_*st*_ is a measure of differentiation between two populations. Values range from 0 (no difference between populations) to 1 (fixed differences between populations). Stemming from Sewall Wright's guideline, *F*_*st*_-values between 0 and 0.05 indicate “no to little genetic differentiation” while *F*_*st*_-values between 0.05 and 0.15 represent moderate differentiation. *F*_*st*_-values between 0.15 and 0.25 are considered large and *F*_*st*_ values above 0.25 show a very large degree of genetic differentiation (Wright, 1978; Balloux and Lugon-Moulin, 2002; Tishkoff and Kidd, 2004; Elhaik, 2012; Bhatia et al., 2013). Researchers consider *F*_*st*_ of 0.25 as a minimum value for genetically distinct races (Templeton, 1998; Graves, 2011).

Another way to measure population differentiation is by using statistical cluster based methods. These methods seek to group individuals together who are genetically similar. Clusters may be defined by calculating pairwise distance matrix and identified graphically. Or model based methods could be used. These models require *a priori* specification of model parameters including the number of clusters. Both types of approaches are utilized in current population structure software packages such as *Eignstrat* (genetic distance) and *Structure* (model based). In this paper, we will focus on results using the computer program Structure. *Structure* uses a Bayesian cluster analysis approach where the researcher arbitrarily determines the number of K clusters into which the data should be portioned (Bolnick, 2008). If the number of clusters is not known, *Structure* allows researchers to define the interval of the values of K from 1 to an arbitrary number *N* (1 ≤ *K* ≤ *N*) and then to compute the maximum likelihood of K-clusters in order to determine the most supported K within the defined interval. However, Evanno et al.'s (2005) *ad-hoc* second order statistic ΔK does not allow *K* = 1 (Evanno et al., 2005; Schwartz and McKelvey, 2009).

### F_*st*_ does not support the existence of distinct clusters

Genomic research is continually providing an improved understanding of factors affecting population differentiation. Take for example the study by Rosenberg et al. (2005). In this study, *F*_*st*_ was modeled as a function of geographic distance (*D*) and other barriers (*B*) (Rosenberg et al., 2002). *D* is a continuous measures while *B* = 1 if a barrier but zero otherwise. After examining pair-wise *F*_*st*_ between individual populations the following regression equation: *F*_*st*_ = 0.0032 + 0.0049*D* + 0.0153*B* was generated. This equation suggests that the Sahara, Himalayas and oceans introduce genetic discontinuities between pairs of populations on the opposite side (*R*^2^ = 0.0153). Crossing any of these barriers amounts to traveling over 3100 km on the same side of the barrier. That is, barriers so different in nature and settlement history add each nonetheless the same value to the *F*_*st*_. However, geographical distance (or isolation by distance; IBD) explains the bulk of the variance (*R*^2^ = 0.690). Yet according to the SHO hypothesis, it is the abrupt tiny increase in the *F*_*st*_ values (*R*^2^ = 0.0153), putatively caused by spatial resisting forces, that enables computer algorithms to partition humans into continental genomic clusters (Rosenberg et al., 2005; Rosenberg, 2011). Nonetheless, using multilocus genetic markers, about 93% of the total human genetic variation was found at the individual level while an *F*_*st*_ of 4.3% was apportioned to “continental” regions (Rosenberg et al., 2002).

Yet even if one aims lower on the aformentioned scale of genetic differentiation, it is still clear that the *F*_*st*_-value of 0.043, measuring the genetic difference between continental clusters (Rosenberg et al., 2002), unambiguously lies in the interval of no to little degree of differentiation on Wright's guideline. Continental subpopulations are also very similar and do not reach, any meaningful degree of differentiation in Darwinian classification. These results suggest that human races, understood as continental clusters, have no taxonomic meaning that warrants granting them an objective biological existence. Actually, the *F*_*st*_-value is even lower, 0.036, with a partition scheme that identified 7 DL-2 meta-populations (Rosenberg et al., 2002). Nevertheless, although generally below the threshold of taxonomic meaningfulness, *F*_*st*_-values, particularly pair-wise *F*_*st*_, vary and are influenced by many factors (Lewontin, 1972; Barbujani et al., 1997; Jorde and Wooding, 2004; Tishkoff and Kidd, 2004; Al Sweih et al., 2010; Graves, 2011; Elhaik, 2012). For instance, data from the International HAPMAP consortium estimated differences among a limited number of selected continental populations to be between 0.11 and 0.19 (Nelis et al., 2009).

The problem is that taxonomic groupings that map actual evolutionary relationships among populations in a worldwide comparison identify series of meta-populations different from continental clustering schemes (Zhivotovsky et al., 2003). Pair-wise *F*_*st*_ computations confirm for example that genetic distances between sub-Saharan African “hunter and gatherers,” the first series of meta-populations (hereafter S_1_-Metapopulations), and sub-Saharan African “farmers” (S_2_-Metapopulations) are greater than between the latter and Europeans (Zhivotovsky et al., 2003; Tishkoff et al., 2009; Kalinowski, 2010). In addition, some populations in the S_1_-Metapopulations such as “the southern Bushmen, central forest Pygmies, and the Hadza compared with Europeans, have *F*_*st*_ estimates in excess of 0.23, approximately twice the average *F*_*st*_ between other global populations” (Henn et al., 2011, 2012). Even so, “East Africans” and the Maasai are more similar to Europeans than to the KhoeSan populations. Moreover, within the S_1_-Metapopulations, the Sandawe for example, are more similar to Europeans than they are to the Hadza (Henn et al., 2011, 2012). Actually, genetic patterns from all four modes of human inheritance (mtDNA, Y-chromosome, X-linked and autosome), along with protein markers, showed that continental clustering represents no natural classification of humans (Maglo, 2011; Mersha and Abebe, 2015).

Be that as it may, the emerging scientific consensus is that while isolation by distance explained a large proportion of human population pair-wise *F*_*st*_-values, cluster, the computational placeholder for race, explained <2% (Rosenberg et al., 2005; Handley et al., 2007). Thus, the indistinctiveness *F*_*st*_-value argument, as construed here against the idea of biological reality of human races, is not simply about crude *F*_*st*_ measures. It also takes into consideration the part of *F*_*st*_ quantitatively explained by cluster/race (Table 2). So it goes beyond Wright's qualitative guideline about the use of *F*_*st*_. The argument thus has two quantitative components, the unadjusted *F*_*st*_-values and the adjusted values of cluster/race covariate. It can thus be considered the quantitative equivalent of the qualitative argument of “lack of distinction” Darwin used to question the taxonomic wisdom of categorizing humans into races in natural classification since the categories cannot be objectively defined (Maglo, 2011).

**Table 2:** **Hypothetical pair-wise ***F***_***st***_ values with corresponding adjusted values of IBD and cluster/race (using 77% for IBD and 2% for cluster)**.

**Pair-wise *F_*st*_***	**Covariates**	
	**Isolation by distance (IBD)**	**Cluster/Race**
0.01	0.0077	0.0002
0.05	0.0385	0.001
0.1	0.077	0.002
0.15	0.1155	0.003
0.2	0.154	0.004
0.25	0.1925	0.005
0.3	0.231	0.006

*Race typically is a confounder whose statistical value becomes vanishing when relevant covariates are known and controlled for. It has no natural reality but more or less an instrumental function depending on the context of our scientific knowledge*.

The above considerations underscore the claim that “cluster” is likely a byproduct of isolation by distance and sampling procedures (Serre and Paabo, 2004; Handley et al., 2007; Schwartz and McKelvey, 2009). For instance, geographic discontinuous samples of Africans, Europeans and East Asians yield clustered representations of the datasets. But when South Asian samples are included in the analyses, clinal representations emerge (Bamshad et al., 2001; Jorde and Wooding, 2004; Tishkoff and Kidd, 2004). The sampling of geographically isolated populations has been called “island model” sampling procedure (Bamshad et al., 2004; Maglo, 2011). It produces a misleading representation of the human genetic continuum (see Figure 1A).

### Cluster based methods, underlying assumptions and the instrumental status of continental cluster/race

There are two different statistical models within *Structure*. One assumes uncorrelated allele frequencies and one assumes correlated allele frequencies. As early as 2004, critics pointed out that the SHO hypothesis failed to be confirmed when one combines an uncorrelated frequencies model with a sampling strategy that assumes a continuous geographic dispersal of human populations. The uncorrelated model yielded a clinal, rather than a clustered, representation of human population genetic structure (Serre and Paabo, 2004). The model used by Rosenberg et al. (2002, 2005) assumes that allele frequencies between continental clusters are correlated, due to common ancestry. Actually, the statistical model of correlated allele frequencies assumes a sharing of a recent common ancestor and admixture between human populations. However, the admixture assumption is contrary to the criteria for biologic natural reality of phylogentic classification. Furthermore, both the correlated and the uncorrelated models of *Structure* converge in showing empirically that human populations are not monophyletic groups (Serre and Paabo, 2004; Rosenberg et al., 2005).

An examination of the assumptions behind the correlated and uncorrelated allele frequencies models will yield insight into the controversy about the biological meaning of continental clusters. The “historical” context of the dispute is that the uncorrelated model was implemented in the computer program *Structure* earlier while the correlated allele frequencies used by Rosenberg and his colleagues was a later revised model (Pritchard et al., 2000; Falush et al., 2003). From an epistemic perspective, the novelty in the revised model was to distinguish between two conditions described as “harsh prior” and “permissive prior.” It is the harsh prior model that assumes that allele frequencies are statistically dependent and hence very similar. So the allele frequency distribution for one cluster provides information about the frequency distributions of the other clusters. The permissive prior makes no such assumptions and population movement is not subjected to the “unrealistic” condition that all subpopulations simultaneously drifted away from a common ancestral population (Falush et al., 2003).

Now, while the harsh prior of statistically dependent frequencies model is described as more efficient at detecting finer population substructures and best suited for “subtle admixture problems,” the authors of the revised *Structure* model also stated that “if the values of *F*_*k*_ are being used to make evolutionary inferences, a permissive prior is more appropriate” (Falush et al., 2003). Additionally, they warned about a crude attribution of biological meaning to variance partitions, including K-clusters with the highest probability. As they put it: “(1) it is computationally difficult to obtain accurate estimates of Pr(X/K), and our method merely provides an *ad-hoc* approximation, and (2) the biological interpretation of K may not be straightforward” (Pritchard et al., 2007). The question then is about how to interpret the *F*_*st*_ values (*F*_*k*_ in the program *Structure F* model) in clustering analysis of human genetic variation.

Furthermore, it has been shown that the rate of individuals having membership in multiple clusters increases with the inclusion of admixed populations in studies. This does not however negate the computational possibility of clustering admixed individuals. But under this scenario, many individuals will typically have mixed membership in different clusters (Pritchard et al., 2007; Bryc et al., 2010; Maglo, 2011; Jin et al., 2012). As mentioned above, the correlated allele model was specifically designed to resolve “subtle admixture problems.” Curiously, some researchers perform cluster analysis on admixed populations by bypassing this model (Tang et al., 2005), raising questions about their findings (Graves, 2011). Yet the user guide of *Structure* states that “Admixture is a common feature of real data, and you probably won't find it if you use the no-admixture model” (Pritchard et al., 2000; Elhaik, 2012).

The admixture-based argument holds indeed that, in a global partition of human genetic variation, the number of individuals with membership in multiple clusters will increase with the inclusion of more admixed populations, causing continental clusters to dissolve into a cline (Bamshad et al., 2003, 2004; Maglo, 2011). Admixture does not however occur only in a demographic melting pot situation. Neighboring-mating likely plays a role in the dissolution of cluster into cline in the “island model” cases discussed above. Neighboring-mating engenders local genetic autocorrelation which is shown to impact strongly the determination and reliability of clusters (Schwartz and McKelvey, 2009).

Spatial genetic autocorrelation results from proximal mating between individuals at the periphery of a geographically dispersed population and neighbors from the surrounding populations. The authors of *Structure* acknowledged the limitations of the program in this respect by stating that in the case of datasets structured by IBD, “allele frequencies vary gradually across the region. The underlying structure model is not well suited to data from this kind of scenario. When this occurs, the inferred value of K, and the corresponding allele frequencies in each group can be rather arbitrary. Depending on the sampling scheme, most individuals may have mixed membership in multiple groups” (Falush et al., 2003; Schwartz and McKelvey, 2009). In a word, computational success does not by itself alone entail the natural reality of clustered entities in evolutionary classification (Maglo and Martin, 2012).

### Continental clusters as computational artifacts

A study on short tandem repeats (STRs)/microsatellite markers from the Diversity Panel dataset demonstrated that continental clusters masks evolutionary relationships among human populations. Estimates of divergence time (*T*_*D*_) showed that S_1_-Metapopulations (see above) were the first series of meta-populations to split from the common ancestral human population. S_2_-Metapopulations, the second series of African meta-populations, and “non-Africans” split from each other at a later time. Principal Component Analysis showed S_1_-Metapopulations at the edge of the sub-Saharan African cluster. The authors consequently wrote: “Each of the large population groups (sub-Saharan African farmers, Eurasia, and East Asia) can be considered as a metapopulation consisting of populations with some genetic exchange between them and with a common ancestry. This is suggested by the value of the statistic S4, which is substantially greater for the pooled regional groups than for single populations within those regions…” (Zhivotovsky et al., 2003). But the pooled statistical value (0.89) of three S_1_-Metapopulations' samples was very close to their individual values (0.85). This suggests, under the hierarchical structure model, that they do not come from one single meta-population. In fact, S_1_-Metapopulations provide crucial information about human origin and evolutionary history (Zhivotovsky et al., 2003; Henn et al., 2012; Veeramah et al., 2012).

**Figure 2:**
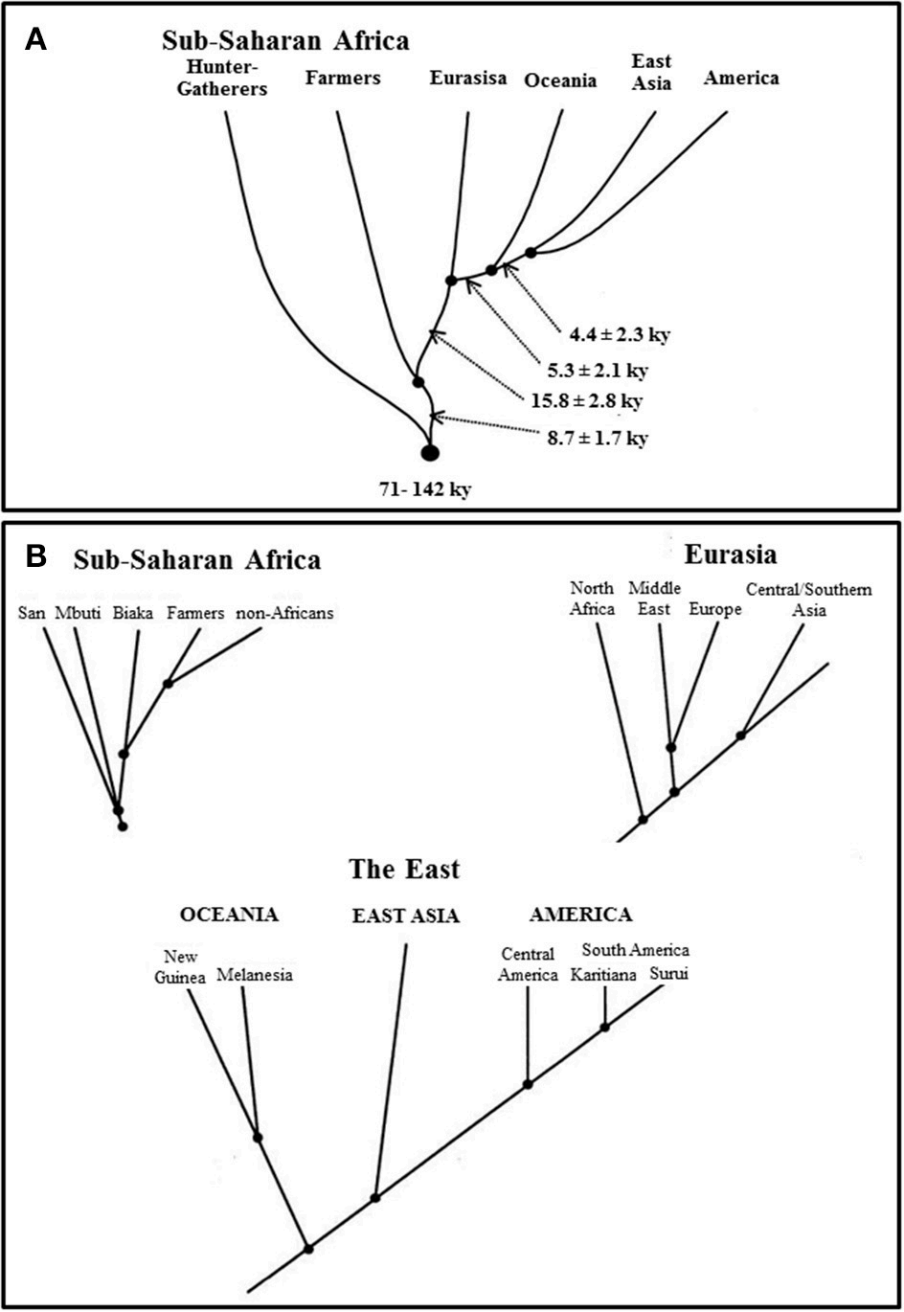
**Human metapopulations based on their divergence time estimates and evolutionary relationships (adopted from Zhivotovsky et al., 2003, Figure 5)**. Zhivotovsky et al. (2003)'s Figures 5A,B clearly indicate that human metapopulations with evolutionary implications do not correspond to continental clusters considered as the placeholders for race in human population genomics. [“Population tree based on *T*D estimates of divergence time. **(A)**. Divergence among major groups. The time estimates are based on 374 STRs (three outlying STRs with tetranucleotide repeats were omitted). Arrows indicate the time (lower bounds, in ky) between adjacent nodes, assuming a generation length of 25 years. **(B)**. Schemes of divergence within the major groups, based on the 374 STRs. Time estimates within each continental group were omitted, because they may be biased owing to possible differential gene flows from other groups”] (Zhivotovsky et al., 2003). Figure reproduced with permission from Cell Press/Elsevier.

**Figure 3:**
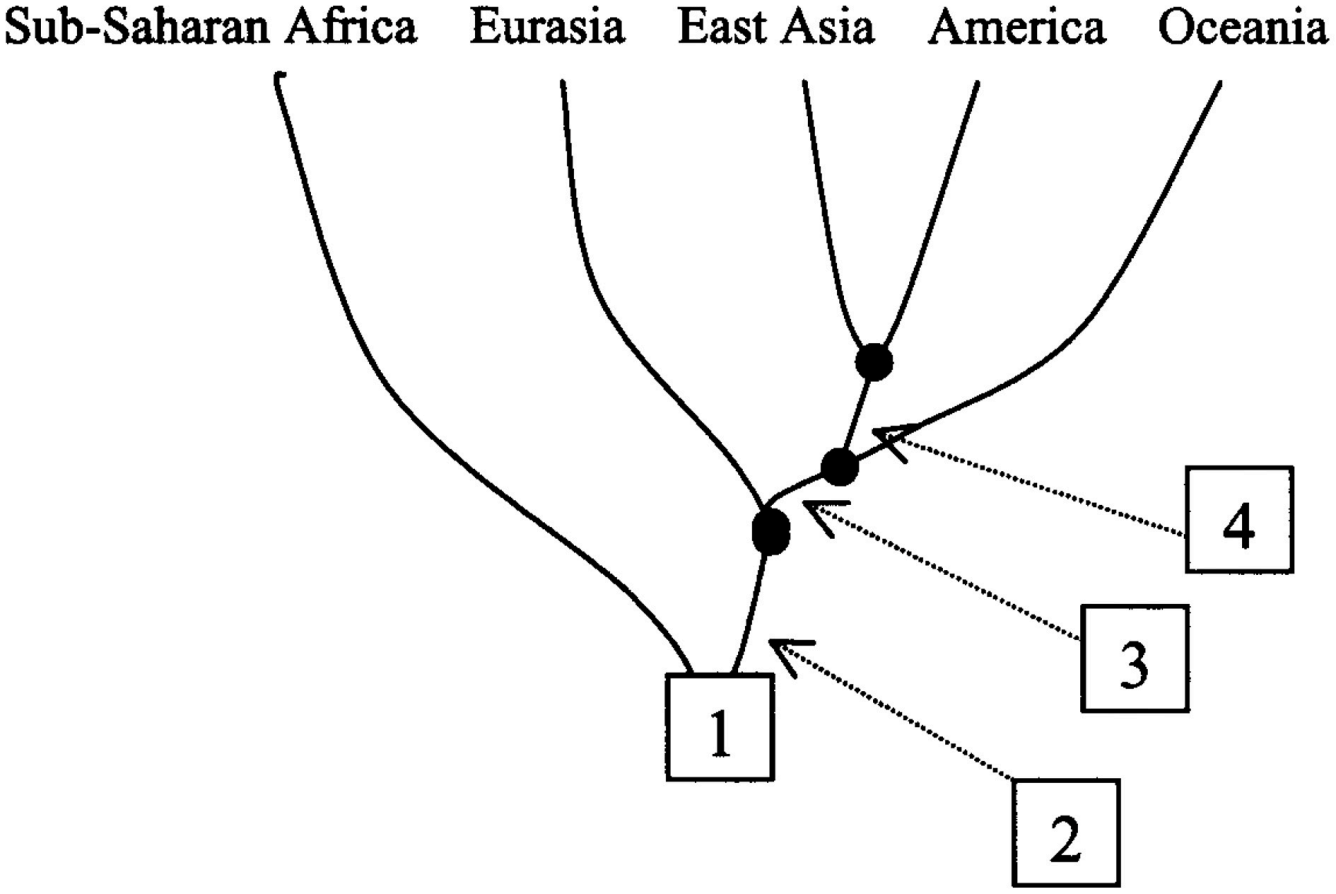
**Conveniently defined metapopulations that mask human evolutionary history and relationships (adopted from Zhivotovsky et al., 2003, Figure 6)**. This tree is simply instrumentally produced to meet the demand of our interest in continental groups/human races and may function under some circumstances as useful problem-solving tool. However, from an evolutionary perspective, it carries no natural meaning or independent reality. [“Reduced population tree showing four separation events”] (Zhivotovsky et al., 2003). Figure reproduced with permission from Cell Press/Elsevier.

This study thus showed not only that there are many sub-Saharan African DL-2 meta-populations at a global level (Figure 2B) but also that S_2_-Metapopulations are evolutionary closer than their neighbor S_1_-Metapopulations, to “non-Africans” (Figure 2A). Nevertheless, this study illustrates also how continental clusters may be instrumentally produced with real data. In fact, when the study goals were altered, the researchers were able to reduce the evolutionary tree by considering sub-Saharan African populations pragmatically “as a single group” (Zhivotovsky et al., 2003). The reduced tree then helped generate a continentally clustered picture of the dataset, a picture that masks the fragmentation history and the evolutionary relationship among human populations (Figure 3).

It is noteworthy that Rosenberg et al. used *Structure* to study the same data set of (STRs)/microsatellite markers as Zhivotovsky et al. (Rosenberg et al., 2002; Zhivotovsky et al., 2003). But *Structure* identified six clusters, with the 6^th^ comprising the Kalash from Pakistan. In 2005, the 6^th^ cluster sometimes subdivides Native American populations across runs (Rosenberg et al., 2005). In 2011, the Oceania cluster was fully identified only at *K* = 6 (Rosenberg, 2011). That is, the number of K-clusters that allegedly corresponds to major geographic regions varies from 5 to 6 with the same data set and computer algorithm. Yet although the features of the SHO hypothesis fully emerged in Rosenberg's work (Rosenberg, 2011), this study was in short of addressing the emerging finding about the complex genetic diversity between African populations and non-African population. Complex genetic diversity which, in the meantime, also received support from Y-chromosome studies (Cavalli-Sforza, 2000; Ingman et al., 2000; Yu et al., 2002; Underhill and Kivisild, 2007; Kalinowski, 2010; Henn et al., 2011; Maglo, 2011).

In Tishkoff et al.'s (2009) study, *Structure* identified 14 clusters within our species (Tishkoff et al., 2009). That is, there are presumably fourteen DL-2 meta-populations worldwide rather than 5. Interestingly, in this study, at *K* = 5, most populations in S_1_-Metapopulations split from S_2_-Metapopulations (Tishkoff et al., 2009). In 2011, Rosenberg also reported the same split but at *K* = 6 in the ninth run (Rosenberg, 2011), indicating that the continental cluster storyline, together with the SHO hypothesis, may be falling apart. Actually, the authors of *Structure* warned against the dogmatization of any given value of K for geographically dispersed populations like humans by explaining that estimations of K work well only for “data sets with a small number of discrete populations” and that in the case of “real-world data sets” structured by IBD or inbreeding “there may not be a natural answer to what is the “correct” value of K” (Pritchard et al., 2007).

There is, however, more. As explained above, our evaluation focuses primarily on studies using the method of the software *Structure*. Yet, in current population genomic research, methods such as Principal Component Anlysis (PCA) are frequently used to study population substructure and to determine ancestry (Li et al., 2008; Crosslin et al., 2014). PCA, like the Baysian approach of *Structure*, is a powerful tool in styuding ancestry and population structure. Generally speaking, population structure analysis plays a very useful role in understanding the relatedness among humans and their ancestral origins as well as for designing disease genetic studies (Baye, 2011; Baye et al., 2011). Ancestry informative markers (AIMs) are tremendously important in this respect because they help understand population history and probe genetic and non-genetic disease susceptibility factors and treatment response determinants (Galanter et al., 2012; Ricks-Santi et al., 2012; Hollenbach et al., 2015). However, it is important to note that the selection of AIMs will influence the clusters generated with common variants detecting more continental ancestry while rare variants detect different patterns (Baye et al., 2011). Thus, when racial categories are predicated on ancestral membership identifications, it is necessary that the issue pertaining to their putative objective natural reality be assessed based on the evolutionary criteria of common ancestry and degree of similarity regardless of the method used to apportion genetic diversity and determine ancestral groupings.

### Spatiotemporal factors and the theoretical meaning of continental clusters

Over the past two decades, genomic research has increasingly supplied various types of empirical evidence which, within an evolutionary framework, unambiguously refutes claims about the natural reality of human races. For instance, by 2002, it had become clear, as discussed above, that genetic distance is greater among sub-Saharan Africans than between some sub-Saharan African meta-populations and “Eurasians” regardless of whether one uses mtDNA, X-linked or autosome genetic markers (Ingman et al., 2000; Yu et al., 2002; Zhivotovsky et al., 2003; Maglo, 2011). Cavalli-Sforza referred to the pattern of genetic distance between Africa and Europe as an “anomaly.” Actually, the Africa-Europe anomaly (hereafter AE-A), consists of: AE-A_1_—an unexpected shortness of genetic distance between sub-Saharan Africa and Europe compared to sub-Saharan Africa and other continents (Cavalli-Sforza, 2000; Tishkoff and Kidd, 2004); AE-A_2_—some meta-populations in sub-Saharan Africa being genetically more similar to Europeans than to neighboring meta-populations on the same side of the Sahara (Ingman et al., 2000; Yu et al., 2002; Kalinowski, 2010; Henn et al., 2011). Yu et al. enunciated what we construe as AE-A_2_ with the revealing title “Larger Genetic Differences within Africans than between Africans and Eurasians” (Yu et al., 2002).

A point that needs to be emphasized here is that not only are there many sub-Saharan African DL-2 meta-populations at a global level (Figure 2B) but also that S_2_-Metapopulations are evolutionary closer than their neighbor S_1_-Metapopulations, to “non-Africans” (Figure 2A). An evolutionary-based grouping of world populations attempts to summarize the complex human population history (Figures 2A,B) while an instrumental grouping lumps pragmatically world populations into five continental groups reducing evolutionary relations (Figure 3). It is this instrumentally engineered clustered picture of human evolutionary history that is misleadingly construed as corresponding to socially defined races in countries such as the US. Although these socially defined races and continental genetic clusters do not actually match, the alleged correspondence has generated its own sets of debates (Maglo, 2010; Maglo et al., 2014).

Yet perhaps, spatiotemporal correlates (IBD and divergence time) are the factors underpinning continental clusters because they appear to comprise populations whose ancestral origins are close in space and time regardless of genetic dissimilarity. Simulations using *Structure* suggest that, at a constant degree of differentiation, cluster membership varies with sample size and divergence times (Kalinowski, 2010). Spatiotemporality is crucial to the evolutionary ordering of living things. Descent with modification (generation) and adaptation (environment) are just some of the familiar evolutionary concepts that implicitly deploy spatiotemporal parameters (Mayr and Bock, 2002). Yet the spatiotemporal proximity of ancestral origins of continentally dwelling subpopulations simply reveals the storing functionality of the cluster construct instead of the ontological order of a natural classification.

By natural classification, we mean, in Duhem's sense, the theoretical organization of experimental laws in a given scientific domain such that they reflect “real relations among things” (Duhem, 1991). Duhem considered scientific theories as a means to logically classify experimental laws. These laws depict symbolical relations between phenomena but not the intrinsic nature of things (Duhem, 1991). Nonetheless, “the more complete” a scientific theory “becomes,” Duhem wrote, “the more we apprehend that the logical order in which theory orders experimental laws is the reflection of an ontological order, the more we suspect that the relations it establishes among the data of observation correspond to real relations among things, and the more we feel that theory tends to be a natural classification” (Duhem, 1991: 26-7). Duhem's model of natural classification is zoological classification. The zoologist, he maintains, considers the genealogical relations established among animals to reflect a natural order in such a way that even if evolutionary theory happens to be proven false s/he will “continue to believe that the plan drawn by his classification depicts real relations among animals; he would admit being deceived about the nature of these relations but not about their existence” (Duhem, 1991).

That said, we distinguish questions of “reality” from questions of “utility” (Maglo, 2007, 2010, 2012). In the not too distant past, determining continental ancestral origin was an astounding achievement. Today, however, the geographic origin of an individual can be determined within just a few hundred kilometers (Novembre et al., 2008). Indeed, with genetic data we are able to subdivide even relatively homogeneous countries into sub-national genomic groups corresponding to linguistic affiliations, e.g., Switzerland (Novembre et al., 2008) or to North-Central-South geographic location, e.g., Sweden (Salmela et al., 2011). However, in a rational classification of biological organisms, the computational possibility to determine group membership (Edwards, 2003; Edge and Rosenberg, 2015) does not imply that these groups are meaningful according to biological systematic and evolutionary classification criteria (Cavalli-Sforza, 2000; Bolnick, 2008; Maglo, 2011). Thus, it is essential that the utility of these classifications are carefully evaluated in well-controlled epidemiological and clinical contexts (Maglo, 2010, 2011, 2012; Maglo and Martin, 2012; Mersha and Abebe, 2015).

## Implications and the potential utility of cluster/racial grouping

As we are transitioning from the universalist clinical concept of “one dose fits all” toward personalized precision medicine, attention has been called to the implications of the phenomenon of phenotypic plasticity in stratified medicine because of the environmental correlates of epidemiological and pharmacogenomic profiles (Maglo and Martin, 2012). Studies have indeed shown great variability in the distribution and expression of clinically relevant genetic variants across subpopulations within continents due to various evolutionary and environmental mechanisms, including ecological and socio-cultural factors (Wilson et al., 2001; Burroughs et al., 2002; Bains et al., 2013). As an increasing number of researchers have shown, it is important for the success of personalized precision medicine that human genetic diversity be considered (Lu et al., 2014; Petersen et al., 2014). But a continental level substructure or race may very well be a confounder in epidemiologic and clinical research. For instance, race accounts for 14.2% of the variance in warfarin dosing when not considering other factors. Yet when pharmacogenomic and relevant biomarkers are taken into account, the statistical value of race was markedly attenutated, 0.3% (Kahn, 2013). This indicates that, from a clinical genomic perspective just as from evolutionary and population genomic perspectives, race is a notion that has at best a contextual instrumental value (Maglo, 2010, 2011).

Despites its lack of natural ontological character, cluster may well be a useful subsidiary notion alongside the foundational concept “cline” in human population genomics. Moreover, because of ecological and environmental variability, a wastebasket taxon may be useful in population health studies. Genetic polymorphisms influencing disease incidence and drug response in humans vary among individuals but they also show patterns of geographic distributions (Baye et al., 2009; Bains et al., 2013). Examples of functional variants exhibiting clear geographic distributions include the ABO blood system (Cavalli-Sforza et al., 1994), sickle cell anemia (Piel et al., 2010) and cystic fibrosis (Bobadilla et al., 2002). However, continental genetic clusters are determined primarily by use of large sets of neutral markers. While a single neutral marker may be in linkage disequilibrium with disease susceptibility or treatment response locus, this single marker is not however sufficient to define populations. Furthermore, susceptibility variants and drug metabolizing enzymes are not the focus of genomic diversity studies, thus making it difficult to generalize the relationship between population diversity on a genomic scale (e.g., across many markers) and risk variants. Nonetheless, as mentioned in the introduction, the frequency of many health-related phenotypes exhibit variation by race. For example the rates of breast cancer are highest in individuals of European ancestry and lowest in individuals of Asian ancestry, with African American in between (Howlader et al., 2014b). However, diagnosis of breast cancer at an early stage age was less common in African Americans while survival was lowest in African Americans and highest in Asian Americans (Iqbal et al., 2015). These differences in outcomes may be due in part to underlying genetic differences as African Americans have higher rates of triple negative tumors, which are known to be more aggressive and require different therapeutic approaches (Howlader et al., 2014a). As such, epidemiologic evidence showing racial differences in health outcomes means that public health and clinical interventions need to consider race.

What this suggests is that researchers and clinicians need to approach race with caution both in the lab and in clinics. The extent to which continental genomic clusters provide useful actionable information to biomedicine remains an open question. Recent studies showed that ancestry mapping has been successfully applied for disease in which prevalence is significantly different between the ancestral populations to identify genomic regions harboring diseases susceptibility loci for cardiovascular disease (Tang et al., 2005), multiple sclerosis (Reich et al., 2005), prostate cancer (Freedman et al., 2006), obesity (Cheng et al., 2009), and asthma (Vergara et al., 2009). Yet, the problem is that even when self-report racial/ethnic identity are said to correspond generally speaking to continental genetic ancestry, racial/ethnic descriptors in the US, for instance, are not necessary good indicators of the complex history of an individual's genetic ancestry (Mersha and Abebe, 2015). The genetic make-up of individuals are highly variable but can however be captured with large dimensional genomic data. Emerging technologies now make it possible to genotype hundreds of thousands of genetic variations in individuals, across the genome with great potential in biomedical research. It is the understanding of the complexity of human individual genetic ancestry that is bringing us closer to personalized medicine (Madore et al., 2007; Bielinski et al., 2014).

It is also important to note, that race is a construct with social and cultural underpinnings that may have biological and biomedical implications. In the United States there are for example marked differences between African Americans and European Americans with respect to economic factors such as education, income, rates of poverty, and rates of being on public insurance (Elster et al., 2003; Williams, 2005; Adler et al., 2012; Cheng et al., 2015). In addition, there is still marked segregation nationally and even within communities (Cable, 2013) which may contribute to health disparities. Thus, simply attributing differences in population groupings to differences in underlying biology may be short sided and may actually cause more harm than good. For example, African Americans have higher rates of obesity than whites (Ogden et al., 2012; Romero et al., 2012). However, there are many potential reasons for increased risk of obesity which may be separate from underlying biology, including local environment and availability of healthy food options and cultural food preferences. Indeed, in Africa the rates of obesity are much lower than what is reported in African Americans in the US (Maglo and Martin, 2012; Mersha and Abebe, 2015). Without understanding the complex dynamic between biologic differences and socio-cultural factors, the optimal strategies for reducing obesity risk cannot be determined and may be missed. Thus, the authors caution against the generalization of the importance of race without by neglecting other factors which may be at play.

## Conclusion

It is important in race debate for researchers to distinguish between pragmatically useful and natural biological classifications. Just because we can identify continental ancestral membership computationally does not necessarily imply that race as defined by continental ancestry is meaningful in biological systematics and evolutionary classification. In fact, the genomic and statistical evidence currently available shows that phylogenetic and genetic similarity-based concepts of race fail to be applicable to humans even under minimal rational theoretical principles currently accepted in population genetics/genomics. Although spatiotemporal parameters connect evolutionary and environmental medicine, continental clusters may not necessary be the most relevant partitions in biomedicine. Awareness of the mere instrumental function of cluster and race by researchers and practitioners is necessary to avoid the reification and naturalization of these notions in the lab and clinic. Epidemiological research and pharmacogenomics indicate indeed that biomedical and statistical values of race are generally vanishing when relevant covariates are controlled for.

## Author contributions

All authors listed, have made substantial, direct, and intellectual contribution to the work, and approved it for publication.

### Conflict of interest statement

The authors declare that the research was conducted in the absence of any commercial or financial relationships that could be construed as a potential conflict of interest.

## Abbreviations

SHO, Sahara: Himalayas and Oceans
DLs: Divisionary Levels
DL-1: Divisionary Level 1
DL-2: Divisionary Level 2
IBD: Isolation by Distance
S_1_-Metapopulations: First Series of (sub-Saharan African) Meta-populations
S_2_-Metapopulations: Second Series of (sub-Saharan African) Meta-populations.
